# The Influence of Monomer Composition and Surface-CrossLinking Condition on Biodegradation and Gel Strength of Super Absorbent Polymer

**DOI:** 10.3390/polym13040663

**Published:** 2021-02-23

**Authors:** Jung Soo Kim, Dong Hyun Kim, Youn Suk Lee

**Affiliations:** 1 Department of Packaging, Yonsei University, Wonju 26493, Korea; kimjungsoo11@kitech.re.kr; 2 Human Convergence Technology R&D Departments, Korea Institute of Industrial Technology (KITECH), Ansan 15588, Korea; dhkim@kitech.re.kr

**Keywords:** superabsorbent polymer, biodegradable, surface-crosslinking, itaconic acid, cellulose

## Abstract

In this study, a superabsorbent polymer (SAP) comprising poly (IA-co-cellulose-co-VSA-co-AA; ICVA) core-SAP (CSAP) was synthesized through radical polymerization using itaconic acid (IA), acrylic acid (AA), cellulose, and vinyl sulfonic acid (VSA) as monomers. The absorption performances and relative biodegradability of various compositions prepared by adjusting the amounts of cellulose and VSA with constant IA and AA content were compared. Increasing the cellulose content in CSAP contributed to improved biodegradation of the surface-crosslinked SAP (SSAP) and gel strength, although the free absorbency (FA) and centrifuge retention capacity (CRC) decreased. Increasing the VSA content resulted in strong anionicity, which enables the absorption of large amounts of water. Surface-crosslinking technology was applied to the CSAP synthesized with the optimal composition ratio to increase its absorption performance and gel strength. Improved performance of the synthesized SSAP (a CRC of 30.4 g/g, absorbency under load (AUL) of 23.3 g/g, and permeability of 55 s) was achieved by selecting the optimal surface-crosslinking treatment time and the amount of distilled water in the surface-crosslinking solution: as the latter was increased in the surface-crosslinking solution, the AUL and permeability of the SSAP were improved, and its biodegradability was found to be 54% compared to the 100% biodegradable cellulose hydrogel in the control group.

## 1. Introduction

A superabsorbent polymer (SAP) can absorb water at tens to hundreds of times its own weight and does not easily release water under a certain pressure. Due to these characteristics, they are most commonly used in sanitary products such as diapers for infants and adults, absorbent pads and packaging materials for food distribution, water-retaining soil and seedling sheets in agriculture and horticulture, and drug delivery materials for pharmaceuticals [1,2,3].

Recently, many studies have been conducted to improve both permeability and absorbency under load (AUL) by increasing the gel strength while maintaining other water absorption performance for application to infant disposable diapers. Normally, commercial SAPs used for disposable diapers have a centrifuge retention capacity (CRC) of about 30 g/g and AUL of about 20 g/g when measured using the EDANA (European Disposables and Nonwovens Association) method [4]. The gel strength can be enhanced by increasing the density of the internal and surface-crosslinking. However, increasing the gel strength narrows the network width and reduces absorption capacity at the same time. In this trade-off relationship, it is important to select the appropriate monomers and find the optimal polymerization conditions [5,6,7,8].

SAPs, which show excellent absorption characteristics, are generally synthesized using petroleum-based monomers such as acrylic acid (AA) or acrylamide. However, they have disadvantages such as being toxic to the skin and are environmentally unfriendly [9,10]. Accordingly, many researchers have studied the synthesis of SAPs using bio-based monomers to reduce the content of petroleum-based monomers such as AA and acrylamide [11,12,13,14,15,16]. Typically, potato starch, cassava starch, corn starch, and cellulose have been widely used in biomass-based SAP synthesis because these are inexpensive and widely available. Replacing SAP production using petroleum-based monomers with starch-based monomers can reduce the final cost and improve the biodegradability of the materials. Biomass-based SAP has excellent biodegradation properties, but has limitations in absorption performance such as gel strength. Accordingly, biomass-based SAP has poor performance as a diaper material, and has mainly been used as an agricultural material.

Itaconic acid (IA), which is often used as a biodegradable monomer, is an unsaturated dicarboxylic acid that is stable at room temperature and easily degrades into soil because it is prepared by cultivating bacteria in sugarcane badges. Furthermore, IA can be radically polymerized with vinyl monomers. Therefore, it can replace petroleum-based AA. Moreover, IA has two carboxylic acid groups and is water-soluble, and it has been selected by the US Department of Energy as the “most valuable substance in biomass” [17]. Since IA can be used as a component in SAPs, it can be used to create new biomass-based polymers that can mitigate some environmental problems [18].

Cellulose is a natural polysaccharide formed by plant photosynthesis and provides biological properties such as biocompatibility and nontoxicity [19]. Cellulose can improve hydrophilicity due to its hydroxylic groups, which can also contribute to improved gel strength [20,21]. However, most biomass-based SAPs have low gel strength, so their uses in biopharmaceuticals and agriculture are limited [22,23,24].

Vinyl sulfonic acid (VSA) is known to be hydrophilic and has high absorption performance. It has been reported that VSA is beneficial for gel expansion by inducing a reaction force between the polymer chains through its negatively charged sulfonate groups [25]. Accordingly, VSA has been widely used as a comonomer in copolymerization studies to improve SAP performance [26,27,28].

The most frequently applied technology in SAP studies to increase gel strength is surface-crosslinking. This produces additional networks bonded to a solid surface by reacting with functional groups that have survived on the core-SAP (CSAP) surface. The aim of surface-crosslinking technology is to enhance the strength of a gel when it is absorbing large amounts of liquid, and various parameters can be adjusted to improve its performance [29,30,31]. Gasri et al. reduced the surface-crosslinking treatment time of SAPs using a microwave method, studied the effects of *N*,*N*-dimethylaniline as a catalyst, and compared their performances according to the type and amount of surface-crosslinking agent [32]. Ha et al. produced AA-based SAPs containing different amounts of ethylene glycol diglycidyl ether and measured the thicknesses of their surface-crosslinked layers using a fluorescent dye [33]. Lee et al. compared the performances of AA-based SAPs according to surface-crosslinking time and the amount of polyethylene imine used as a surface-crosslinking agent: the thickness of the surface-crosslinked layer increased, and the absorbency decreased with increased surface-crosslinking time [34].

In this study, we confirmed the change in absorption properties according to the monomer composition and surface-crosslinking conditions in order to prepare SAP that has biodegradability and the performance required for diapers. Biomass-based IA and cellulose were used to enable biodegradation, and VSA having strong polarity and AA having excellent hydrophilicity were used as monomers to complement the absorption performance [35,36]. There is no study on an IA and cellulose combined SAP, which has better absorption performance, compared with existing eco-friendly SAP. The surface-crosslinking density of the synthesized CSAP was further strengthened by esterification between hydroxide carboxyl groups on its surface and hydroxide groups on the surfaces of the surface-crosslinking agents. In addition, optimal conditions were determined by adjusting the surface-crosslinking treatment time and the amount of distilled water in the surface-crosslinking solution, after which the absorbency, gel strength, permeability, and biodegradability were evaluated.

## 2. Materials and Methods

### 2.1. Materials

Itaconic acid (IA, Junsei, special grade, Tokyo, Japan) and vinyl sulfonic acid (VSA, Sigma Aldrich, St. Louis, MI, USA), acrylic acid (AA) were used as a monomer without purification. Cellulose (CMC, Daejung, M_w_: 21,000–500,000, density: 1.59, Siheung, Korea) was purchased and used without purification. Poly(ethyleneglycol) diacrylate (PEGDA, Sigma Aldrich), 1,6-hexanediol diacrylate (HDODA, Sigma Aldrich) were used as the inner-crosslinker, sodium persulfate (NaPS, Sigma Aldrich) was used as the initiator, and 1,4-butanediol (BD, Sigma Aldrich, 99%) was used as the surface-crosslinker without purification.

### 2.2. Preparation of the CSAP

IA, cellulose, VSA, and AA were added to the total content of 100 g in a four-neck reactor and 80 g of distilled water was added to maintain a nitrogen atmosphere (Table 1). A 50% NaOH aqueous solution was slowly added to the reactor until it comprised 70% of the total molar ratio of total monomers. After the temperature was stabilized to less than 50 °C, 0.2 g of HDODA and PEGDA were added as inner-crosslinker and mixed uniformly. Thereafter, 0.5 g of NaPS was added to the reactor and stirred for 2 h. Then, the reaction was transferred to a plastic tray and dried in a vacuum oven at 60 °C for 24 h. The dried product was frozen in a −20 °C freezer and then pulverized with a grinder. The crushed product was spread evenly on a wide tray and dried at 60 °C for 12 h. The dried product was classified into a particle size of 300 to 600 μm. We showed synthetic composition of CSAP and abbreviation of SAP made of poly(IA-co-cellulose-co-VSA-co-AA; ICVA).

### 2.3. Preparation of the Surface-Crosslinked SAP (SSAP)

Table 2 shows the composition of the surface-crosslinking solution and surface-crosslinking time for the preparation of SSAP. Solvent (methanol + distilled water) and BD were mixed in a ratio of 10:1 to prepare a surface-crosslinking solution in a 20 mL vial. CSAP (3 g) was added to the surface-crosslinking solution and swelled for 10 min. After removing the surface-crosslinking solution, the swollen CSAP was transferred to an aluminum tray and reacted at 170 °C for 15.0–25.0 min.

### 2.4. Measurement

#### 2.4.1. Fourier-Transform Infrared (FT-IR) Spectroscopic Analysis

The structure of SAP was analyzed by Fourier-transform infrared spectroscopy with a resolution of 0.5 cm^−1^ (FT-IR, Nicolet iS10, Thermo Fisher Scientific, Waltham, MA, USA) using potassium bromide (KBr) pellets in the scan range of 400–4000 cm^−1^.

#### 2.4.2. Free Absorbency (FA)

SAP (0.1 g) was placed in a 100 mesh tea bag and swelled in excess distilled water at room temperature until the swelling equilibrium was reached. Then, the tea bag was taken out of the distilled water, excess water on the outside was wiped off, and it was weighed. In order to compare the absorption properties of SAP under various conditions, FA was measured in a 0.9 wt% NaCl aqueous solution under the above conditions. A 0.9 wt% NaCl aqueous solution was used according to the international EDANA method (NWSP 240.0.R2), which has similar composition to human urine. FA is calculated as follows:

(1) $$FA=\frac{\omega_{1}-\omega_{0}}{\omega_{0}}$$

where *ω*_1_ and *ω*_0_ are the weights of the swollen and dried SAP, respectively [37].

#### 2.4.3. Gel Content

SAP (0.5 g) was immersed in 500 mL DW and stirred for 48 h to extract unreacted monomers and oligomers. After that, the SAP was screened and dried in an oven at 60 °C until there was no change in weight. The gel content is calculated as follows:

(2) $$Gel\;content=\frac{\omega_{e}}{\omega_{i}}\times 100$$

where *ω_i_* and *ω_e_* are the weight of initial and extracted dried SAP, respectively [38].

#### 2.4.4. Centrifuge Retention Capacity (CRC)

CRC is a value indicating how much water is retained after dewatering the swollen SAP with a centrifuge. SAP (0.1 g) was swollen in 0.9 wt% NaCl aqueous solution for 30 min then dehydrated to 300 G in a centrifuge and weighed according to EDANA method (NWSP 241.0.R2). CRC is calculated as follows:

(3) $$CRC=\frac{\omega_{2}-\omega_{1}}{\omega_{0}}$$

where *ω*_1_ and *ω*_0_ are the weight of the swollen and dried SAP, respectively.

#### 2.4.5. Absorbency Under Load (AUL)

**Figure 1:**
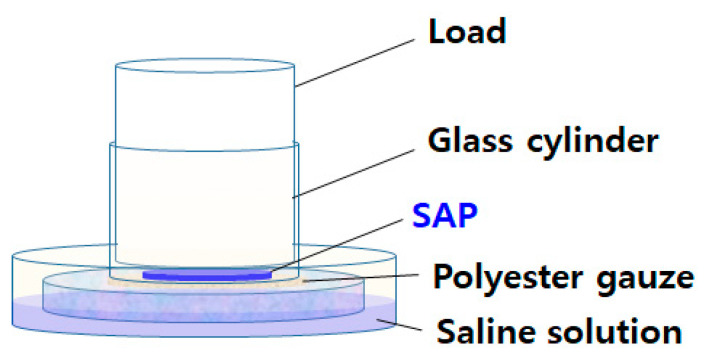
Scheme of the absorbency under load tester.

AUL is a value that indicates how much moisture the SAP can absorb under a given pressure. It is caused by the pressure applied to the SAP due to the infant’s activity, and the average value of the load is taken as 0.3 psi according to EDANA method (NWSP 241.0.R2). As in the measurement of free absorbance, 200 mL of distilled water was poured into a beaker, and NaCl was added to prepare a 0.9 wt% NaCl aqueous solution. A total of 0.16 g of the SAP was evenly distributed in the prepared cylinder, and the weight was taken using 0.3 psi weights. Then, a ceramic filter plate was placed on a chalet, and filter paper was placed on it. The cylinder containing SAP was placed on the filter paper, and sufficient aqueous solution of NaCl was added. After 1 h, the cylinder was taken off, and the weight was measured with the weights. AUL is calculated as follows:

(4) $$AUL=\frac{\omega_{2}-\omega_{1}}{\omega_{0}}$$

where *ω*_1_ and *ω*_0_ are the weight of the swollen and dried SAP, respectively (Figure 1).

#### 2.4.6. Permeability

Permeability is a measure of the amount of water flowing between swollen SAP layers. First, 0.5 g of SAP was added to the equipment and SAP was swollen for 30 min by adding a sufficient amount of 0.9 wt% NaCl solution. A 0.3 psi pressure device was installed on the swollen SAP. The cock at the bottom of the device was opened and the time taken for the 20 mL 0.9 wt% NaCl solution to pass was measured.

#### 2.4.7. Rheological Properties

The rheological measurements were performed using a rotational rheometer (TA instrument Ltd., ARES-G2, New Castle, DE, USA) with a parallel plate geometry (plate diameter of 25 mm and a gap of 3 mm). The SAP sample (0.2 g) was added to 10 mL of DW and swelled for 30 min. The rheological properties were measured at 25 °C after placing the expanded gel particles on a parallel plate of a rheometer. The storage modulus (G′) and loss modulus (G″) were recorded at constant shear strain (0.2%) and a frequency ranging from 0.1–100 Hz [39].

#### 2.4.8. Degree of Biodegradation

Permeability is a measure of the amount of water flowing between swollen SAP layers. First, 0.5 g of SAP was added to the equipment and SAP was swollen for 30 min by adding a sufficient amount of 0.9 wt% NaCl solution. A 0.3 psi pressure device was installed on the swollen SAP. The cock at the bottom of the device was opened and the time taken for 20 mL of the 0.9 wt% NaCl solution to pass was measured. For the degree of biodegradation test, the percentage biodegradation of ICVA SAPs were investigated according to ISO 14851:1999 and Chung-ang university laboratory method [40]. The microorganism solution was prepared by mixing solid solution diluted with standard media. Each sample, after freezing and drying, was mixed with microorganism solution in a BOD bottle. All sample bottles were kept in an incubator at 25 °C.

The degree of biodegradation was calculated as follows:

(5) $$Biodegradation\;\left(\%\right)=\frac{BOD_{s}}{ThOD}\times 100$$

where *ThOD* is the theoretical oxygen demand in mg/g of test material, *BOD_s_* is the specific BOD in mg/g of test material, as shown in Equation (6):

(6) $$BOD_{s}=\frac{BOD_{t}-BOD_{b}}{P_{t}}.$$

*BOD_t_* is the BOD of the flask containing the test material in mg/L, *BOD_b_* is the BOD of the blank flask in mg/L, and *P_t_* is the concentration of the test material in the flask in mg/L.

## 3. Results and Discussion

### 3.1. Synthesis of ICVA CSAP

**Scheme 1:**
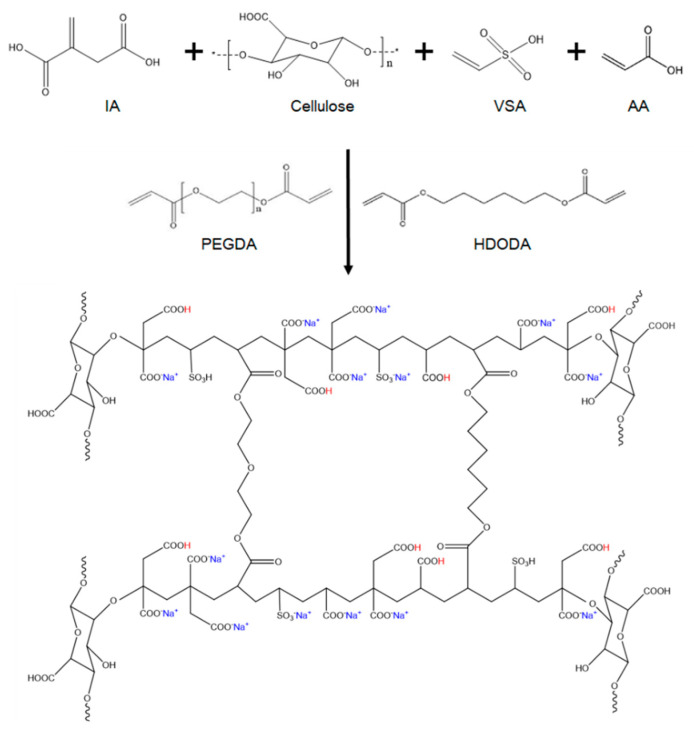
Schematic synthesis of CSAP.

For the CSAP synthesis, various monomers such as IA, cellulose, VSA, and AA were mixed in the water and added an internal crosslinking agents and initiator (Scheme 1). IA, VSA, and AA are highly hydrophilic materials due to hydroxyl groups, and radical polymerization is possible because of vinyl groups. The hydrogen atoms in the hydroxy groups in cellulose contribute to the formation of more active alkoxy radicals, while radical anions dissociated from the ammonium persulfate (APS) initiator react with vinyl monomers to form polymer chains, in which crosslinking occurred due to the internalized crosslinking agent. The monomer composition ratio of IA and AA was fixed at 50:40 and the ratio of cellulose and VSA was adjusted at the remaining 10%. A total of 0.5 g of 1,6-hexanediol diacrylate (HDODA) and poly (ethylene glycol) diacrylate (PEGDA) at a 1:1 ratio as the surface-crosslinking agent was added along with 0.5 g of APS as the initiator (Table 1).

### 3.2. FT-IR Analysis of Monomers and IC_2.5_VA CSAP

**Figure 2:**
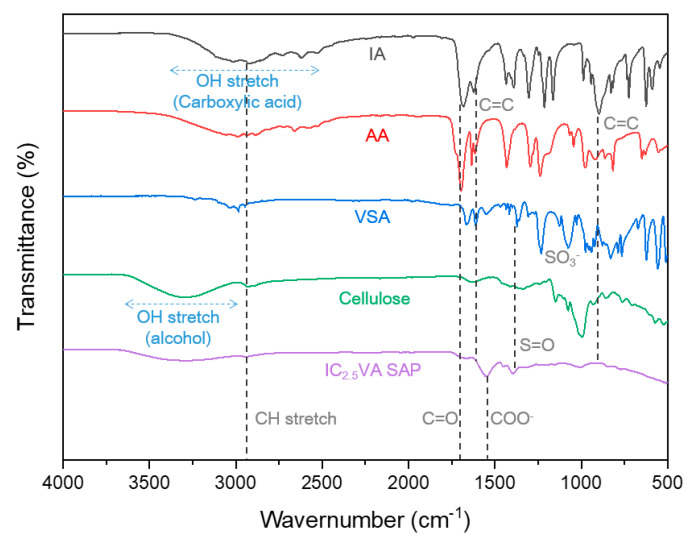
FT-IR spectra of IA, cellulose, VSA, AA, and IC_2.5_VA CSAP.

Figure 2 presents Fourier-transform infrared spectroscopy (FT-IR) spectra of IA, AA, VSA, cellulose, and IC_2.5_VA CSAP. The absorption band at 1721 cm^−1^ were attributed to the C=O stretching vibrations of carboxyl groups (–COOH) in IA and AA, respectively. The peak at 819 and 1680 cm^−1^ indicates C=CH_2_ of vinyl groups in IA, AA, and VSA. In IC_2.5_VA-CSAP, the –COO– peak was identified in the region around 1584 cm^−1^ due to the stable resonance effect of the neutralized carboxylic acid. In the spectrum of the cellulose, absorption peaks at 3550–3200 cm^−1^ correspond to O–H stretching, while for VSA, peaks consistent with S=O absorption at 1300 cm^−1^ and –SO_2_–O stretching vibration at 1184 cm^−1^ were evident. In IC_2.5_VA, C–H stretching (the same as in AA and IA) was seen at 2930 cm^−1^, and C–O–C and C–O–H stretching occurred at 1015 cm^−1^. The characteristic peaks of each monomer were observed in the IC_2.5_VA CSAP spectrum. In addition, the C=C peaks appearing in IA, VSA, and AA disappeared by polymerization, which infers the successful synthesis of IC_2.5_VA CSAP.

### 3.3. Properties of SAPs According to Composition of Cellulose and VSA

CRC and AUL values were compared according to CSAP composition (Table 3). Higher content of VSA has better CRC since VSA can absorb a large amount of water due to its high anionicity. Moreover, more cellulose improved the AUL due to its large surface area and porosity in the SAP structure.

**Figure 3:**
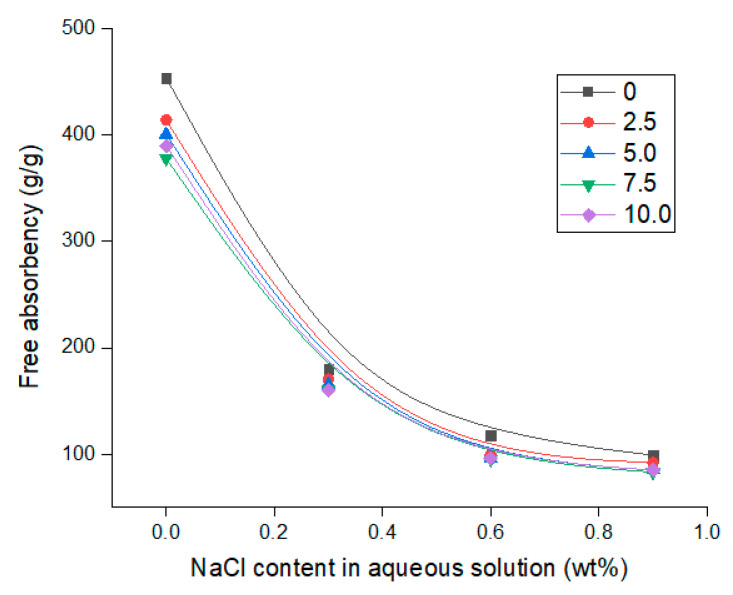
The free absorbency of the CSAP according to content of cellulose on various concentration of NaCl solution.

In Figure 3, the IVA shows high free absorbency (FA) at all concentrations in 0.0–0.9 wt% NaCl solution and 454 g/g in distilled water. IC_2.5_VA containing cellulose presented a high FA of 414 g/g in distilled water and 92 g/g in a 0.9 wt% NaCl solution. Therefore, surface-crosslinking studies were conducted using IC_2.5_VA, which has excellent CRC, AUL, and FA values.

The synthesized ICVA CSAP was treated with surface-crosslinking agent 1,4-butanediol (BD) while mixing with water and methanol to improve the gel strength. Hydroxy functional groups in BD participated in esterification with the residual carboxyl groups in IA, cellulose, and AA. This reaction occurred irregularly on the ICVA CSAP surface and did not have much effect on the inside of the CSAP. However, it is important to select the appropriate amount of water in the surface-crosslinking solution because it is a significant factor in determining the depth at which BD penetrates into the CSAP. Deeper penetration increases the surface-crosslinking density and improves gel strength by reducing the FA value.

Table 4 compares the absorbency of the surface-crosslinked SAP (SSAP) according to the composition ratio of the comonomers. As the cellulose amount in the component ratio increased, CRC decreased while AUL increased. This showed a similar tendency after surface-crosslinking treatment, especially when 2.5 g cellulose was injected, which improved the AUL and permeability. Cellulose can have a positive effect on permeability because it has a large surface area relative to its molecular weight, has excellent dispersion in water, and can give high porosity to the SAP structure. Ali Olad et al. reported that cellulose can provide pores in SAP because it forms physical crosslinking points between polymer chain and grafted polymeric structure. In addition, physical crosslinking density increases in the presence of cellulose due to the generation of additional hydrogen-bonding interactions within the matrix, which results in the formation of stiffer gel network structure with higher gel strength [41]. During surface-crosslinking treatment, the hydroxyl groups of cellulose can form a more robust surface through esterification, which can have a positive effect on AUL, permeability, and gel content. Moreover, the SSAP had 90% good gel content, although no tendency was prevalent in the composition.

**Figure 4:**
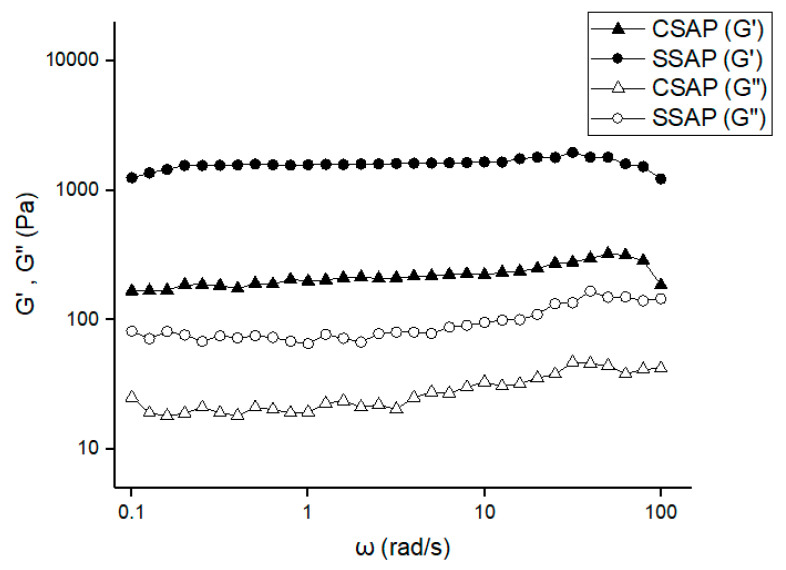
G′ and G″ frequency of IC_2.5_VA CSAP and SSAP.

We measured the storage modulus and the loss modulus in order to compare the gel strength of CSAP and SSAP using IC_2.5_VA samples. Flory-Rehner et al. suggested that the crosslinking density of a polymer is directly related to its storage modulus [38]. As shown in Figure 4, we confirmed that the storage modulus of IC_2.5_VA SSAP was significantly higher than IC_2.5_VA CSAP. Accordingly, we were able to predict that the gel strength of CSAP was improved by surface-crosslinking.

**Figure 5:**
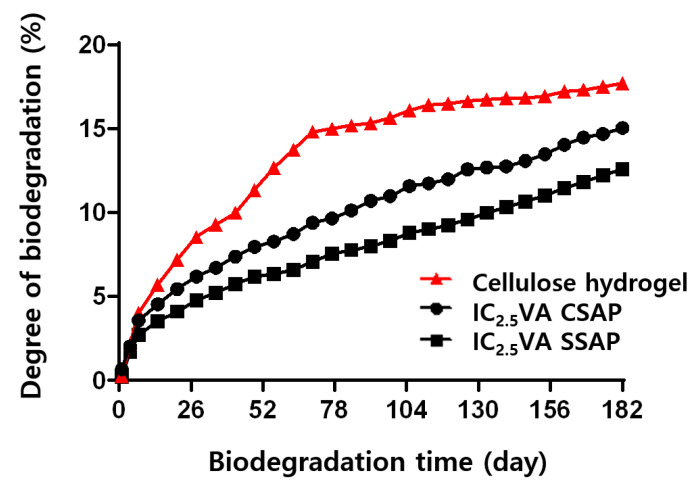
Degree of biodegradation of IC_2.5_VA CSAP and SSAP.

Figure 5 exhibits the results of a comparative study into the biodegradability of IC_2.5_VA and a hydrogel manufactured from 100% biodegradable cellulose as the comparator. The results confirm that CSAP was more easily biodegraded than SSAP. When compared to the relative biodegradability of cellulose hydrogel (based on 100%) after 182 days (26 weeks), CSAP and SSAP had biodegraded by 85% and 71%, respectively. Hence, the increase in crosslinking density by the surface-crosslinking treatment reduced biodegradability, although it was still acceptable. Using IC_2.5_VA, which showed excellent biodegradability and absorbency in the previous experiments, the performance changes were additionally compared with the surface-crosslinking time and solution composition.

### 3.4. Properties of SAPs According to Surface-Crosslinking Time and Composition of Surface-Crosslinking Solution

**Figure 6:**
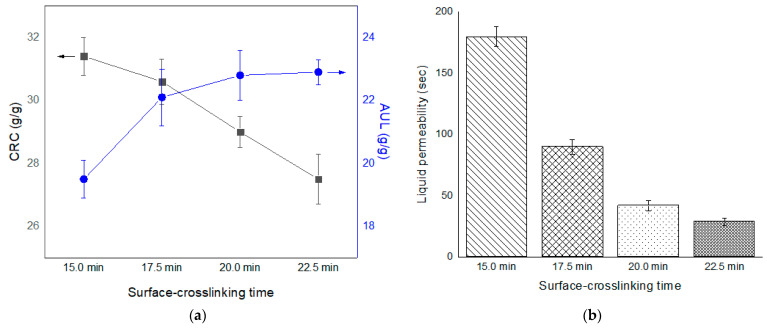
CRC and AUL (**a**) and permeability (**b**) of the SSAP according to surface-crosslinking time.

In the conditions reported in Table 2, the performance of CRC, AUL, and permeability over surface-crosslinking time are compared in Figure 6. The ICVA CSAP synthesized in this study was very sensitive to temperature changes because of it having the highest amount of IA, which has low thermal resistance in the composition ratio. Temperatures above 170 °C or more than 25 min of surface-crosslinking time caused degradation in gel performance due to the thermal decomposition of CSAP. As the surface-crosslinking time increased, CRC decreased whereas AUL and permeability performance increased. In particular, excellent CRC values and decent AUL and permeability values were obtained at 17.5 min of surface-crosslinking time. According to Lee et al., the surface-crosslinked layer thickens as the surface-crosslinking time increases [34]. Nevertheless, the AUL and permeability values could be improved by changing the surface-crosslinking thickness by adjusting the amount of water in the surface-crosslinking solution.

**Figure 7:**
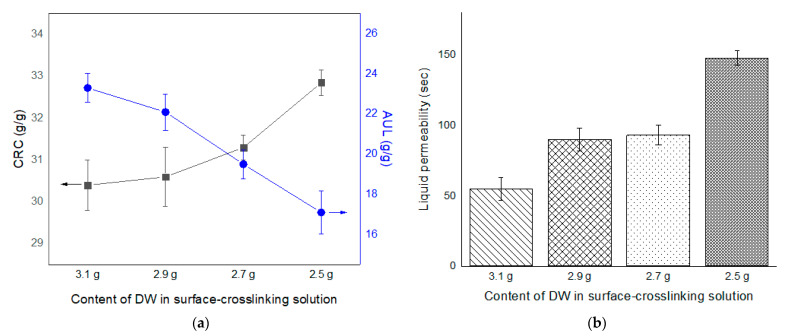
The CRC and AUL (**a**) and permeability (**b**) of the SSAP according to content of DW in surface-crosslinking solution.

Distilled water in the surface-crosslinking solution penetrates the surface of the CSAP. Increased thickness of the surface-crosslinked layer can reduce absorption performance in terms of CRC and FA because it interferes with the expansion of CSAP. When the distilled water content of less than 2.7 g was injected, the CRC value was above 36 g/g but the AUL and permeability values were drastically reduced (Figure 7). On the other hand, when the distilled water content was 3.1 g, CRC was more than 30 g/g, AUL was 23 g/g, and the permeability was 55 s, which are results that indicate excellent performance.

**Figure 8:**
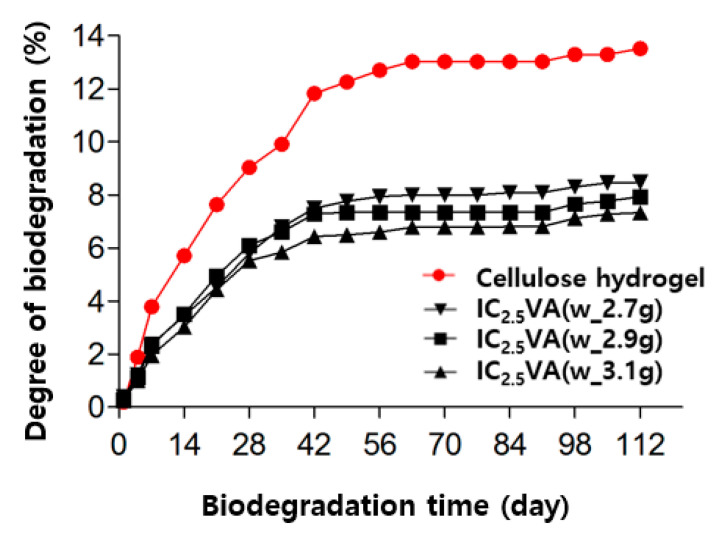
Degree of biodegradation according to water content in surface-crosslinking solution.

In Figure 8, the biodegradability of CSAP is compared with the cellulose hydrogel (the reference specimen) over 112 days (16 weeks) with the distilled water content in the 2.7–3.1 g range. It was found that the crosslinked surface layer was thinner and improved biodegradability as the distilled water content decreased. Compared to the biodegradability of the cellulose hydrogel, IC_2.5_VA (DW_2.7 g) showed a relative biodegradability of 63%, and IC_2.5_VA (DW_3.1 g) showed a relative biodegradability of 54%. The optimum surface-crosslinking treatment conditions for the surface-crosslinking time and the amount of distilled water in the surface-crosslinking solution increased the gel strength by solidifying the surface of the CSAP but reduced the biodegradability. However, this was observing the biodegradation over a relatively short period of time, and when biodegradation was allowed to continue, the improved gel strength of the ICVA coincided with acceptable biodegradability.

## 4. Conclusions

Our research is based on the newly designed eco-friendly biodegradable SAP made of biomass-derived IA and cellulose. The absorption performance of other eco-friendly SAPs is significantly inferior to the existing commercial AA based SAP. On the other hand, we prepared a SAP with almost similar performance of commercial SAP used in hygienic diapers. ICVA CSAP was synthesized using IA, cellulose, VSA, and AA, the successful synthesis of which was confirmed through FT-IR analysis. After surface-crosslinking treatment, the gel content was maintained at around 90%. As a result of adjusting the composition of cellulose and VSA, the FA and CRC of CSAP decreased but the AUL slightly increased as the cellulose component was increased. These results were more pronounced in the SSAP and were particularly effective in improving gel strength. Hence, IC_2.5_VA with appropriate CRC, AUL, and permeability was selected as the optimal composition ratio, and the biodegradability of CSAP and SSAP was compared.

The study of surface-crosslinking treatment time adjustment using IC_2.5_VA confirmed that AUL and permeability improved significantly as the surface-crosslinking treatment time increased. However, the CRC decreased sharply after 20 min, leading to a shorter time (17.5 min) being selected. Furthermore, the performance was supplemented by adjusting the amount of distilled water in the surface-crosslinking solution. When the amount of distilled water was increased from 2.9 to 3.1 g, the AUL and permeability were improved.

It was expected that the increase of the distilled water content in the surface-crosslinking solution facilitated penetration of the surface-crosslinking agent, thereby increasing the thickness of the surface-crosslinking layer.

The successfully developed SSAP with 54% relative biodegradability compared to standard cellulose hydrogel (100% biodegradable), CRC 30.4 g/g, AUL 23.3 g/g, and permeability of 55 s could be a commercial replacement for existing SAPs comprising petroleum-derived raw materials.

## Figures and Tables

**Table 1:** Synthetic composition of CSAP (core-superabsorbent polymer (SAP)).

NAME *	Monomer (g)				Crosslinker (g)		Initiator (g)
	IA	Cellulose	VSA	AA	HDODA	PEGDA	APS
IVA	50	0	10.0	40	0.25	0.25	0.5
IC_2.5_VA		2.5	7.5				
IC_5.0_VA		5.0	5.0				
IC_7.5_VA		7.5	2.5				
ICA		10.0	0				

* Polymerization conditions of CSAP: (a) reaction temperature = 60 °C, (b) reaction time = 2 h, (c) degree of neutralization = 70 mol%, and (d) medium volume = 80 mL.

**Table 2:** Surface-crosslinking conditions.

CSAP (g)	Surface-Crosslinking Solution Composition			Temperature (°C)	Time (min)
	Methanol (g)	DW (g)	Surface-Crosslinker		
			BD (g)		
3.0	6.1	2.9	1.0	170	15.0
					17.5
					20.0
					22.5
	5.9	3.1			17.5
	6.3	2.7			
	6.5	2.5			

**Table 3:** The centrifuge retention capacity (CRC) and absorbency under load (AUL) of the CSAP according to the composition of monomer.

NAME	CSAP	
	CRC (g/g)	AUL (g/g)
IVA	55.8	6.6
IC_2.5_VA	52.3	7.6
IC_5.0_VA	51.3	7.6
IC_7.5_VA	49.8	7.3
ICA	49.6	7.8

**Table 4:** The absorption properties of the surface-crosslinked SAP (SSAP) according to the composition of monomer.

CSAP	SSAP *			
	CRC (g/g)	AUL (g/g)	Permeability (sec)	Gel Content (%)
IVA	30.6	17.4	126	90.0
IC_2.5_VA	29.0	22.8	42	90.6
IC_5.0_VA	27.0	22.9	35	90.1
IC_7.5_VA	25.5	24.1	25	89.2
ICA	24.4	23.6	28	89.4

* SSAP synthesis conditions: (a) CSAP = 3 g, (b) temperature = 170 °C, (c) reaction time = 20 min, and (d) weight ratio of methanol, DW, and BD = 6.1:2.9:1.0.

## Data Availability

Data sharing not applicable.
